# Transactions Texas State Medical Association, 1889

**Published:** 1889-10

**Authors:** 


### Book Notices.

### TRANSACTIONS TEXAS STATE MEDICAL ASSOCIATION, 1889.

This volume has been issued from the press of Eugene Von Boeckmann, Austin. The mechanical execution is up to the high standard of excellence which characterizes all book work done by this establishment; no better could have been done in Chicago. The book contains 340 pages, neatly printed with new Ronaldson type, on heavy S. & S. C. book paper. Five hundred copies were bound in purple cloth, gilt lettered, uniform with the editions of 1885-6, while one hundred and fifty copies were put up in heavy pamphlet covers.

The secretary has received acknowledgements from a number of Health Boards, etc., thanking him for a copy, and all compliment the work. Dr. Jno. H. Rauch, the famous Illinois sanitarian, says, "The work is a credit to the State of Texas."

The "minutes" or proceedings occupy the first sixty-five pages. This is followed by the classical address of the retiring President —Paine—a mine of interesting matter, testifying alike to deep and patient research, and a high order of intellectual cultivation and ability on the part of the author.

The section work is highly creditable to the Association. While there were not so many papers contributed this year as formerly—and part of those were withdrawn by the authors—those which go to make up the body of the book, are creditable to the Association, generally speaking, and are well worth perusal and preservation. Hence the volume is smaller than some of its predecessors. Only four papers placed in the hands of the publishing committee were omitted,—three for causes satisfactory to the committee, and one, which had been read by caption and referred, after which it appeared in print, thus barring its admission to the transactions under resolution. (See page 304.)

Under the Section on Surgery and Anatomy there are twelve papers, all good, some excellent; Section on Practice, etc., five; Gynecology, three; Obstetrics, four; State Medicine and Public Hygiene, two; Ophthalmology, one; Electro-Therapentics, one; total, twenty-eight.

[As a mere enumeration of the papers would be without interest, the list by title is omitted.]

The appendix, which makes seventy-six pages, is made up of the Constitution and By-laws, into which have been incorporated the amendments adopted at San Antonio; the Code of Ethics; the several ordinances and resolutions that have been adopted from time to time; the Charter of the Association, giving articles of incorporation, names of trustees, etc., and the roll of members, including a table of the officers since its organization in 1869. Active members (504) and honorary members, together with a list of the twenty-four auxilliary societies in affiliation with the State Association; and an errata and an index or table of contents.

The first paper in Section on Surgery—"Some Observations on the Possibilities of Preventive Surgery," by Dr. E. J. Ward, is reproduced in this issue of the JOURNAL; it will be found very interesting.

In reviewing this volume the editor of the JOURNAL is handicapped to a great extent. Being the secretary of the Association and chairman of the Publishing Committee, it ill becomes him to discriminate; and, indeed, where so much is excellent, it would hardly be fair to point out some papers for their excellence and fail to mention others, while to quote and criticise all would take more room and time than we have at our disposal;—hence we conclude to let the papers speak for themselves.

Nor can we very well praise our own work,—the getting up of the volume. We let others do so, and we are gratified to say that we already have received most flattering commendations from distinguished members. The book will be a valuable addition to the library of any practitioner; it contains much that is eminently practical and useful.

